# Circadian Rhythms and Lung Cancer in the Context of Aging: A Review of Current Evidence

**DOI:** 10.14336/AD.2024.1188

**Published:** 2026-01-01

**Authors:** Wenhui Xu, Lei Li, Zhendong Cao, Jinghong Ye, Xuyu Gu

**Affiliations:** ^1^Department of Respiration, The Second Affiliated Hospital of Nanjing University of Traditional Chinese Medicine (Jiangsu Second Hospital of Traditional Chinese Medicine), Nanjing, Jiangsu, China.; ^2^Department of Medical Oncology, Shanghai Pulmonary Hospital, School of Medicine, Tongji University, Shanghai, China.

**Keywords:** circadian rhythms, circadian clock, aging, lung cancer, molecualr mechanisms, BMAL1

## Abstract

Circadian rhythm is the internal homeostatic physiological clock that regulates the 24-hour sleep/wake cycle. This biological clock helps to adapt to environmental changes such as light, dark, temperature, and behaviors. Aging, on the other hand, is a process of physiological changes that results in a progressive decline in cells, tissues, and other vital systems of the body. Both aging and the circadian clock are highly interlinked phenomena with a bidirectional relationship. The process of aging leads to circadian disruptions while dysfunctional circadian rhythms promote age-related complications. Both processes involve diverse physiological, molecular, and cellular changes such as modifications in the DNA repair mechanisms, mechanisms, ROS generation, apoptosis, and cell proliferation. This review aims to examine the role of aging and circadian rhythms in the context of lung cancer. This will also review the existing literature on the role of circadian disruptions in the process of aging and vice versa. Various molecular pathways and genes such as BMAL1, SIRT1, HLF, and PER1 and their implications in aging, circadian rhythms, and lung cancer will also be discussed.

## 1. Introduction

The circadian rhythm is an endogenous coordination system that regulates behavior and physiology over 24 hours and copes with environmental changes [1-3]. In other words, it is a system that accommodates variations in sleep-wake cycles and regulates biological systems such as cardiovascular, endocrine, neurological, metabolic, and immune functions [4]. The circadian clock pervades all kingdoms of life and species. It is regulated at cellular and molecular levels by a complex network of genes and molecular pathways, ensuring adaptation to the day-night cycle [5].

The circadian clock operates through a transcriptional feedback loop, consisting of an activator complex comprising BMAL1, CLOCK, and bHLH-PAS proteins [6, 7]. The transcriptional activator complex induces the transcription of downstream genes which in turn feedback to inhibit self-transcription [8]. Whether it is lifestyle, aging, or environment, disruption in the process of the circadian clock will consequently lead to several health issues including changes in homeostasis, the onset of diseases, and other conditions [9-11]. The processes and mechanisms involving the circadian clock are complex, however, understanding these mechanisms can lead to informed decisions and planning of proper therapeutic strategies [12-14].

Biological aging is a complex physiological process that ultimately results in structural and functional decline in the body and organs [15]. Various cellular and molecular mechanisms have been reported in the process of aging including mitochondrial dysfunction, oxidative stress, metabolism dysfunction, telomere shortening, and deregulated autophagy [16]. Globally, the population is aging at a fast pace, with people over 60 years old projected to rise to 22% by 2050 from 11% in 2006 [17, 18]. Advancements in healthcare have boosted life expectancy worldwide, but as the elderly population grows, frailty and morbidity have become significant public health challenges [19]. Therefore, aging is a significant risk factor for chronic diseases such as diabetes, obesity, cardiovascular disease, cancer, and neurodegenerative disorders [20].

The process of aging causes significant changes in the circadian rhythms such as loss of rhythmicity in circulating hormones, cognitive changes, emotional transformation, and a shift towards morning phenotype [21-23]. A common aspect of aging is the shift to an earlier sleep schedule, often earlier than desired [24, 25]. Older individuals also experience more frequent awakenings and a reduction in the deeper stages of non-REM sleep [26-29]. These changes are linked to sleep complaints, with many studies reporting that over one-third of older adults regularly experience early morning awakenings and difficulty maintaining sleep [30, 31]. Studies involving young adults have revealed that even slight shifts in the timing of circadian sleep phases can profoundly impact the ability to maintain uninterrupted sleep throughout the night [32-34]. Thus, age-related modifications in circadian rhythms or their regulation of sleep might be responsible for the changes in sleep timing and consolidation observed with aging [35].

Lung cancer (LC) continues to be the leading cause of cancer-related deaths globally because it often has no symptoms in its early stages and is typically diagnosed at an advanced stage [36]. Today, LC is the most commonly diagnosed cancer, representing 11.6% of all cancer diagnoses. It is also the leading cause of cancer-related deaths globally for both men and women, with a mortality rate of 18.4% among all cancer types [37]. LC can be classified into two key groups based on histological subtype: small-cell lung carcinoma (SCLC) and non-small-cell lung carcinoma (NSCLC). NSCLC accounts for 85% of all LC cases and includes histological subtypes including large cell carcinoma, squamous cell carcinoma, and adenocarcinoma, with adenocarcinoma being the most common subtype [38].

Numerous studies have shown a strong association between cancer and disorders in circadian rhythm. A comprehensive meta-analysis carried out using multiple datasets of cancer transcriptome has shown that the expression of circadian rhythm-associated genes is dysregulated across various tumor types [39]. First, there is a mutual regulatory mechanism shared by both circadian rhythm and cancer genes. The circadian clock modulates a range of tumor suppressor genes and oncogenes, playing a role in tumor development and malignancy [40, 41]. Secondly, the circadian clock also regulates the rhythmic expression of genes related to metabolic and endocrine functions, both of which are crucial in tumor development [42-45]. Disrupted biological rhythms compromise immunity, facilitating tumor immune escape via immune checkpoints and contributing to malignant tumor progression [46-49].

Studies conducted in humans and animals have shown a dysregulated circadian rhythm increases the risk of LC [50, 51]. Logan et al investigated how chronic circadian disruption influenced natural killer (NK) cell function and tumor growth. They found that circadian disruption altered clock gene expression suppressed NK cell cytolytic activity and promoted lung tumor growth [52]. Research has also demonstrated that both physiological and genetic-based disruption of the circadian clock can lead to low survival and elevated risk of developing LC [50]. In this article, we will summarize the existing literature on the aging-related circadian rhythms and their association with LC development and progression. This review will also focus on how aging, circadian rhythm and LC intersect to influence disease progression and treatment outcomes.

## 2. Methodology

### 2.1 Literature Search Strategy

**Figure 1.**
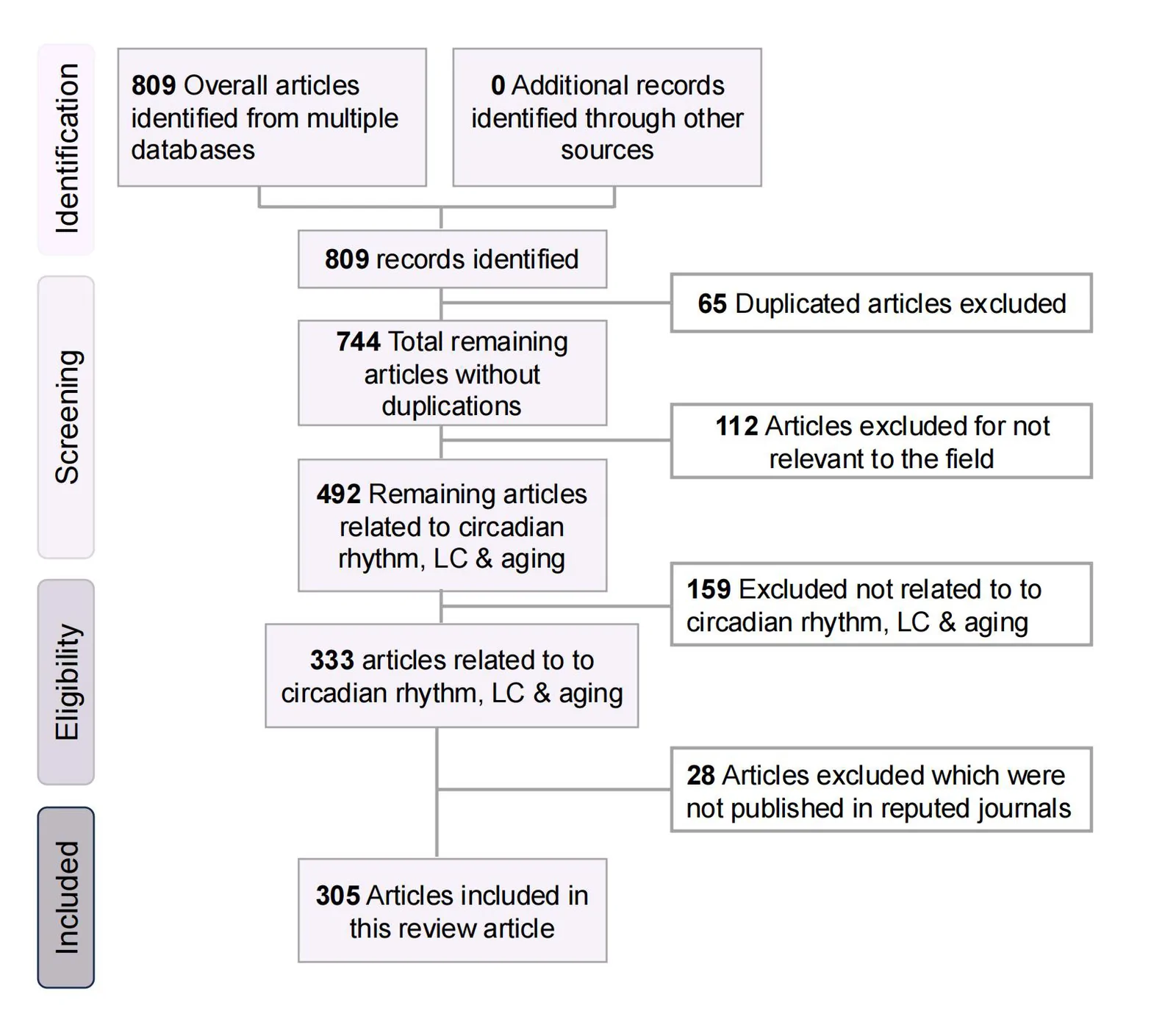
**A flowchart showing the process of article screening for this review article**. A total of 809 articles were retrieved of which 65 articles were excluded being duplicates. From the remaining 744 articles, 140 articles were removed after careful reading of titles and abstracts. Then further articles were excluded which were not relevant to the topic and finally, 305 articles were included in this review.

A systematic literature search was conducted across multiple databases to ensure a comprehensive review of studies related to circadian rhythms, aging, and LC. The databases utilized included PubMed, ScienceDirect, EMBASE, JSTOR, BioMed Central, Medline, and Web of Science. Google Scholar was also used to identify any additional relevant articles. Searches incorporated keywords and MeSH terms related to the core concepts of this review, including “circadian rhythm”, “circadian clock”, “aging”, “lung cancer”, and “molecular mechanisms”. Keyword combinations (e.g., “circadian rhythm AND lung cancer”, “aging AND cancer”) and Boolean operators (AND, OR) were applied to refine the search results. An initial search yielded 809 articles. After removing duplicates, the titles and abstracts of 744 articles were screened. Subsequently, articles that did not meet the inclusion criteria based on relevance or scope were excluded. Full texts of 305 studies meeting the rigorous criteria for inclusion in this review. A flowchart of this process is provided in Figure 1.

### 2.2 Eligibility Criteria

The existing literature on circadian rhythms and LC in the context of aging was searched. Particular focus was given to studies investigating the molecular mechanisms regulating the connection between LC and circadian rhythms. Many articles were excluded, as listed in Table 1.

**Table 1:** Exclusion and inclusion criteria.

No	Exclusion and inclusion criteria
	Inclusion Criteria
**1**	Studies with a clear focus on circadian genes or molecular pathways in relation to lung cancer biology
**2**	Peer-reviewed articles focused on circadian rhythms, aging, and lung cancer
**3**	Studies that provide insights into molecular mechanisms or clinical implications specific to lung cancer
**4**	Research articles that explore the interplay between aging, circadian rhythm disruption, and cancer susceptibility or progression, providing mechanistic insights or clinical relevance
**5**	Exclusion Criteria
	Articles not directly related to the intersection of circadian rhythms, aging, and lung cancer
**6**	Duplicate studies or studies with incomplete data that lack adequate analysis
**7**	Review articles that do not contribute original data or specific mechanistic discussions directly related to lung cancer and circadian rhythm
**8**	Studies with limited methodological rigor, such as those lacking control groups or with small sample sizes that may affect the reliability of findings

## 3. Circadian Rhythm

Almost all living organisms, including humans, have an internal time-keeping system comprising hierarchically organized body clocks. These clocks regulate biological and behavioral rhythms with an approximately 24-hour cycle [53, 54]. This evolutionarily conserved time-keeping mechanism allows organisms to adapt to the constantly changing environment [55]. The circadian clock acts as an automatic regulatory system for the expression, accumulation, and degradation of clock gene products, creating an independent molecular oscillator [56]. Thus, circadian rhythms regulate nearly every aspect of metabolism, from sleep/wake cycles to fasting/feeding behavior, by controlling cellular and organ system functions [57-59].

In the circadian clock, there are two key components, peripheral clocks located in all tissues of the body, and the central clock, located in the suprachiasmatic nucleus (SCN) of the brain [60, 61]. External cues like light signals and feeding rhythms synchronize the central clock, that coordinates the rhythms of peripheral clocks throughout the body [62]. Light enters the eye through the retina, triggering electrical signals that travel along the retinohypothalamic tract and are then converted into chemical signals within the SCN [62]. This internal timing system is thought to improve an organism's chances of survival by enabling it to predict and respond to regular changes in light, food availability, and predation risk. In single-celled organisms, the intrinsic clockwork orchestrates the precise timing of biochemical, metabolic, and redox processes, ensuring the optimal functioning of cell physiology across various species [63].

Studies have established the important role of the circadian system in regulating a range of biological processes including cardiovascular regulation, digestive system, homeostasis, and metabolic system. Disruption of the circadian system has been associated with the development of several conditions and diseases [64-69]. In the context of physiological regulation, redox reactions that involve reactive oxygen and nitrogen species have an important role in governing complex processes such as cellular signaling and immune responses. However, the pathological outcomes of oxidative stress include disturbances in cellular signaling and damaging alterations to critical biomolecules such as DNA, proteins, and lipids [70, 71].

### 3.1 Molecular Mechanisms of Circadian Rhythm

**Figure 2.**
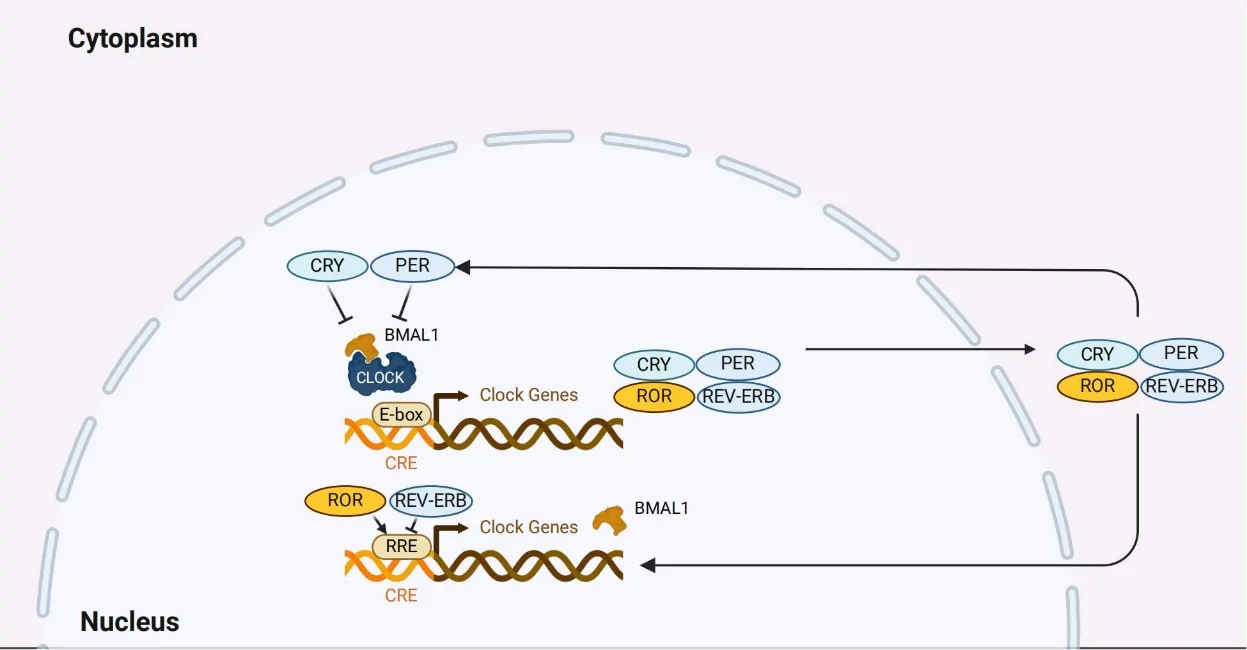
**A visual illustration of CLOCK that involves BMAL1 and CLOCK proteins binding to E-box elements to activate clock-controlled genes (CCGs)**. PER and CRY proteins from Period and Cryptochrome genes inhibit activity. A secondary loop with REV-ERB and ROR regulates BMAL1 transcription.

At cellular and gene levels, the circadian clock is comprised of several sets of genes and transcription factors with autoregulatory loops known as transcription-translation feedback loops (TTFLs). In this loop, the key role is played by the complex resulting from the binding of BMAL1 and CLOCK genes with the Enhancer Box (E-Box)-sequences [72, 73] (Figure 2). BMAL1 and CLOCK increase the expression of other genes such as period (PER proteins) and cryptochrome (CRY proteins). In the cytoplasm, PERs and CRYs produce a complex which is later translocated to the nucleus [74, 75]. Interestingly, research has highlighted the importance of additional transcriptional loops, including PERS proteins repressing their transcription and that of CRYs [76]. It has been also revealed that PER and CRY proteins have CLOCK-independent DNA binding interactions. These interactions are mostly time-dependent but do not involve the CLOCK-BMAL1 complex or other core circadian clock proteins [77].

A decrease in CRY and PER proteins alleviates the BMAL1 activity, allowing a new oscillatory cycle to be established. Beyond this core loop, other important modulators of the circadian clock comprised the nuclear receptors REV-ERBα (NR1D1) and REV-ERBβ (NR1D2), along with the RORα, RORβ, and RORγ [78-80]. These regulators create an additional feedback loop: REV-ERBs function as transcriptional repressors of the BMAL1 gene, whereas RORs enhance BMAL1 expression by binding to the BMAL1 gene promoter [81-83].

The MYC family of proteins, a family of regulation-related genes and proto-oncogenes that produce transcription factors, has been reported to be upregulated in many human cancers [84-86]. Recent findings indicate a compelling bidirectional relationship between MYC and the molecular clock, likely disrupted in various cancers. Multiple studies across different organisms and models have explored how oncogenic MYC affects circadian rhythms, consistently showing that dysregulated MYC disrupts or abolishes the oscillation of molecular clock components. This interaction is illustrated in Figure 1B, where two bodies of research suggest that elevated MYC levels suppress BMAL1, resulting in the loss of oscillatory behavior in molecular clock components [87-90].

Post-translational modifications (PTMs) are essential regulatory mechanisms that finely tune the stability, activity, and function of clock proteins, thereby impacting the precision and robustness of circadian oscillations. Various kinases regulate the functions of several cellular clock proteins, such as CRY1, PER1, CLOCK, PER2, BMAL1, CRY2, PER3 and through phosphorylation [91-93]. The phosphorylated PER proteins are recognized by the F-box protein FBXL3, which facilitates their ubiquitination and subsequent degradation during the early morning and late-night phases of the circadian cycle. The ubiquitination of CRY proteins by FBXL21 regulates their stability and function [94, 95]. Similarly, the acetylation of BMAL1 by the histone acetyltransferase p300/CBP-associated factor (PCAF) increases the transcriptional activity of BMAL1, leading to enhanced expression of clock-controlled genes (CCGs) during the circadian cycle [96, 97].

## 4. The Association Between Aging and Circadian Rhythms

Aging can be seen as a gradual decline in functional capabilities or the deterioration of physiological functions [98]. This inherent, unavoidable, and currently irreversible process increases an organism's vulnerability, leading to a greater loss of viability [99, 100]. Aging could be subdivided into two independents but connected processes. The first process is primary aging which includes the systemic and gradual deterioration of the whole system including body parts, cellular processes, and physiological functions over the years. The second process is secondary aging, also referred to as senescence, which most of the time results from diseases, poor nutrition, lack of physical activities, tobacco and alcohol consumption, and exposure to hazardous materials [101]. Secondary aging is a more “unsystematic” process and how it influences primary aging, is not well explored [102].

The origin of aging has been debated for decades, with numerous hypotheses, mechanisms and evolutions. The leading hypotheses which explain the aging process are categorized into two main groups: programmed theories and damage or error theories of aging [103]. Programmed theories, also known as adaptive theories of aging, suggest that aging is a deliberate process providing evolutionary benefits by limiting lifespan. One example is the mutation accumulation theory, which posits that aging could result from the accumulation of genetic mutations that only manifest in old age, reflecting the reduced influence of natural selection over time [104]. The antagonistic pleiotropy theory on the other hand describes that natural selection favors the harmful genes during the early stages of life [105, 106].

**Figure 3.**
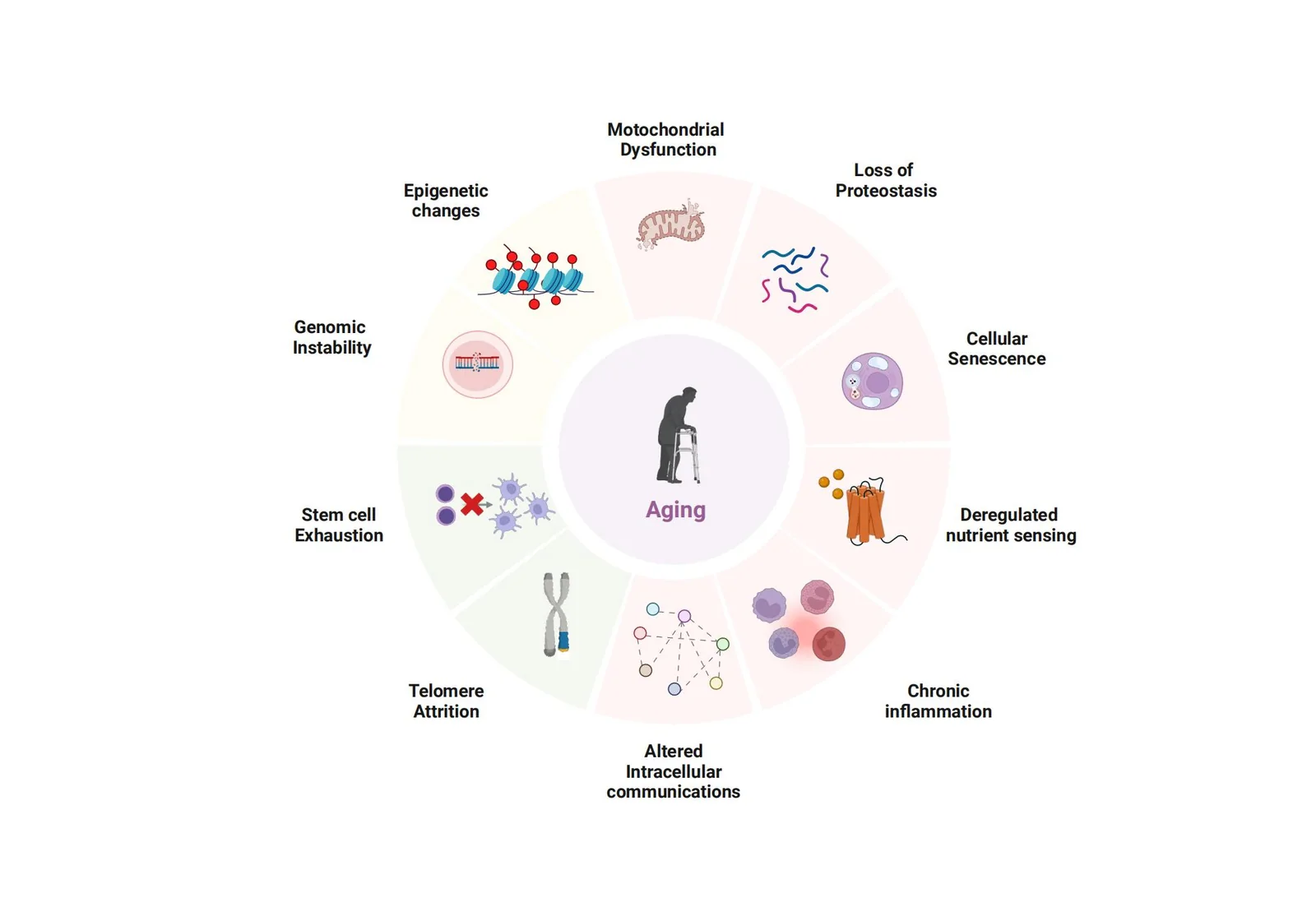
**Key Hallmarks of Aging**. This figure illustrates some of the key hallmarks of aging, including mitochondrial dysfunction, loss of proteostasis, cellular senescence, deregulated nutrient sensing, chronic inflammation, altered intracellular communications, telomere attrition, telomere attrition, stem cell exhaustion, genomic instability, genomic instability, and epigenetic changes.

To understand how aging influences the body, 9 important hallmarks have already been reported by researchers. As shown in Figure 3, genomic instability refers to the increasing mutation rates during the process of aging. The increase may inappropriately express genes that are not favored by the body’s natural process [107]. Other hallmarks of aging are the shortening of the telomeres [108] and epigenetic alterations including histone modifications and DNA methylation [109]. During the process of oxidative phosphorylation, the damage caused by the oxygen radicals is referred to as mitochondrial dysfunction [110] while the increasing inability of aging cells to remove protein debris and misfolded protein is known as loss of proteostasis [111]. Both processes play a key role in the whole process of aging.

Deregulated nutrient sensing disrupts cellular signaling cascades that regulate the balance between rest and metabolic activity [112]. Cellular senescence limits the replicative lifespan of cells and is recognized as a primary driver of aging [113]. Stem cell impairment is thought to contribute to tissue aging, as stem cells are crucial for maintaining tissue homeostasis, and their loss leads to organ dysfunction [114]. Additionally, inflammation and disturbed communication between cells in an organ can cause significant damage to its overall function [115] (Fig. 3).

Several organisms ranging from mammals to microorganisms, have circadian rhythms or circadian clocks. The circadian pacemaker in mammals is located in the brain, a region. A range of organisms, from microorganisms to mammals, have circadian rhythms [116]. In mammals, the circadian pacemaker is present in a region of brain called SCN [117]. The SCN serves as the coordinator and master biological clock center of the peripheral circadian clocks [4]. The SCN controls many processes including rhythmic oscillations through a neuronal pathway which affects melatonin secretion in the pineal gland [118]. The age-induced melatonin reduction could be attributed to the circadian system's failure that is supposed to accurately engage peripheral rhythms. This happens because SCN activity is regulated by the melatonin through a feedback mechanism by the inhibition of its function [119].

Research conducted in animal models has demonstrated that significant changes occur in circadian rhythms during the aging process [120, 121]. This age-induced disruption of the circadian rhythm results in various physiological abnormalities including compromised DNA damage response [122], disturbances in the sleep-wake cycle [123], oxidative damage [124], and reduced activity levels [125]. Studies carried out in humans and rodents have revealed that disruption in circadian rhythms is associated with changes in drinking behavior, body temperature, melatonin secretion, and sleep cycle [126-130]. This highlights the importance of circadian rhythm and its deterioration in the context of aging.

The SCN, as the master circadian clock, regulates various fucntions in synchronizing clocks in peripheral tissues, which change with aging. For example, in older individuals, the lens's reduced ability to transmit light decreases sensitivity to light as a synchronization signal. Additionally, the number of retinal ganglion cells with intrinsic photoreceptors that transmit photic information to the SCN decreases, and the SCN’s overall functioning declines [131, 132]. In the aging SCN, fewer neurons express the neurotransmitter VIP, a crucial modulator of cell-to-cell communication among SCN neurons, compared to the young SCN [133]. Additionally, older SCNs exhibit a reduction in the number of synaptic terminals and altered intercellular connectivity between neurons [134].

In elder people, the biggest argument is the inability to adapt to phase shifts due to circadian disruption. For this argument, Andlauer et al. proposed an explanation that is based on a reduction in circadian rhythm amplitude [135]. This reduction impairs temporal signaling, resulting in the system's inability to synchronize in response to a phase shift. Therefore, a greater amplitude of rhythms would enhance synchronization capability. According to this hypothesis, higher amplitude rhythms improve synchronization capability. However, this adjustment occurs slowly due to the increased amplitude of the rhythms [135].

Evidence from a range of studies have demonstrated that rhythms in the prefrontal cortex (PFC) are crucial for executive function and cognitive performance. Multiple human studies have shown diurnal variations in cognitive performance and a notable decline in performance following disruptions to circadian rhythms [136-138]. Interestingly, these patterns differ by age. Older adults typically reveal better cognitive performance in the morning, with a decline as the day progresses [139]. In older women, weaker circadian activity rhythms are directly linked to poorer executive function [140]. In experiments involving cortical-driven cognitive tasks in mice, researchers observed distinct daily fluctuations in performance [141-143]. Furthermore, mice subjected to a modified light-dark cycle of 20 hours displayed diminished cognitive flexibility and a reduction in dendritic length [144].

In addition to other changes, research has shown age-associated changes in the SCN-regulated rhythms as well [145]. Palomba et al. observed a decline in GABAergic synapses within the SCN in aged mice [146]. GABA-mediated neuronal activity and vasoactive intestinal peptide (VIP) expression are vital for maintaining the synchronization of SCN-associated neuronal firing rhythms [147]. Wang et al. conducted a study on elder participants and reported that disrupted sleep-wake rhythms showed a significant association with the loss of VIP-ergic neurons in their SCN post-mortem examination [148]. Thus, current evidence supports the hypothesis that alterations in SCN neurons may have a greater role in the desynchronization of behavioral rhythms associated with aging. This highlights the increasing significance of SCN neurons in aginging-associated circadian disruption.

Mice that lack BMAL1 provide a good example of how circadian clock proteins influence aging. These mice have been reported to exhibit an increased aging tendency, with an average lifespan of 8 months which is 26 months in wild-type mice. Additionally, mice that are deficient in BMAL1 develop various age-related diseases [149]. Mice that are deficient in clock and PERIOD2 do not exhibit accelerated aging when the conditions are normal. However, when they are exposed to non-lethal doses of radiation, some of the characteristics of accelerated aging do develop in these mice [150, 151].

Studies involving aged animals have demonstrated that PER1 transcription is activated by light and exhibits a notably prolonged resynchronization delay [152]. Conversely, young animals with disrupted Per genes become unresponsive to light cues [153, 154]. Advancing age decreases retinal sensitivity, that leads to a number of age-related diseases and complications. In elderly individuals, homeostatic sleep is linked to the PER3, a circadian clock gene in the coding region. The PER3 gene has been shown to be associated with a phase advance in the melatonin profile, resulting in increased nocturnal wakefulness for the elderly [155]. In situ hybridization analysis for PER2 mRNA showed that the age-associated reduction in diurnal rhythm amplitude in the hippocampus can exacerbate cognitive deficits [156, 157].

The circadian clock’s physiological output is extremely tissue-specific and consists of several key processes and pathways associated with aging [158, 159]. For instance, the physiology of adult stem cells in various tissues exhibits a robust circadian pattern [160]. The circadian clock regulates the cell cycle in stem cell pools in the hair follicle, skeletal muscle satellite cells, hematopoietic stem cells (HSCs) and in basal layer stem cells of the interfollicular epidermis [158, 160-167]. Additionally, the output of circadian in the aged peripheral tissues is reprogrammed, likely contributing to aging.

Research in the last 10 years has highlighted the increasing role of different sirtuins in the regulation of the circadian clock, notably the chromatin-bound nuclear SIRT6, the mitochondrial SIRT3 and cytoplasmic SIRT1 [168]. In aging, the NAD^+^ levels deplete which leads to SIRT1-induced mitochondrial dysfunction [169]. Zhang et al. investigated the role of NAD^+^ and reported that repletion of NAD^+^ improved the stem cell’s function and lifespan of aged mice in an SIRT1-dependent manner [170]. This was validated by another study in which Satoh et al. increased the expression of SIRT1gene in the brains of male and female mice. They reported that the overexpression of SIRT1 led to delayed aging and increased lifespan [171]. These studies suggest the important role of SIRT1 in aging and circadian rhythms.

To summarise, the delicate association between aging and circadian rhythms is evident in the decline of physiological functions and synchronization capabilities over time [172-175]. Aging affects multiple components of the circadian system, including molecular clocks in peripheral tissues and the master clock in the SCN, resulting in disrupted behavioral rhythms and cognitive performance [176-179]. The evidence highlights the significance of preserving strong circadian rhythms to reduce age-related functional and physiological declines [180-188]. As research progresses, understanding the mechanisms behind these changes may offer insights into interventions that could enhance the quality of life in the aging population.

## 5. Aging, Circadian Disruption, and LC

**Figure 4.**
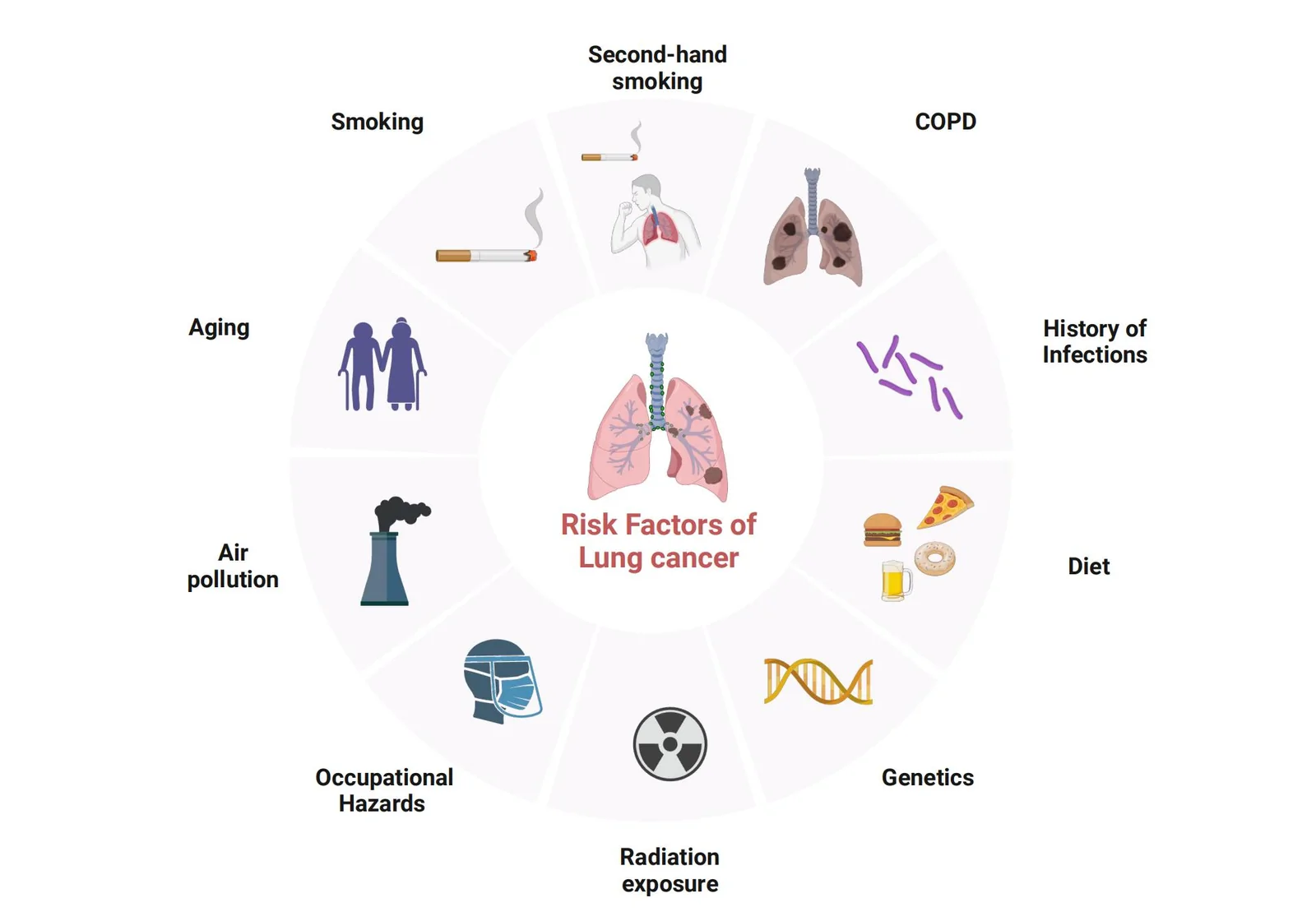
**Key Risk Factors for Lung Cancer**. This visual illustration outlines major risk factors leading to lung cancer, including smoking, chronic obstructive pulmonary disease (COPD), history of infections, diet, genetics, radiation exposure, occupational hazards, air pullution, and aging.

LC ranks as the top cause of cancer mortality globally, with an approximated 2.2 million newly diagnosed cases and 1.8 million deaths occurring in 2020 [189]. LC is primarily categorized into two fundamental types including SCLC and NSCLC. NSCLC is the most prevalent form that constitutes about 85% of all LC cases with further subtypes such as large cell carcinoma, squamous cell carcinoma, and adenocarcinoma. Adenocarcinoma, the most common subtype, typically starts in mucus-secreting cells and often occurs in non-smokers [190-194]. Apart from genetics, smoking, pollution, and biological aging are some of the key risk factors implicated in the development of LC [195, 196](Fig. 4).

In the modern digital world, the disruption of circadian rhythm is quite prevalent due to the increasing use of artificial light use. According to reports, approximately 18-20% of workers in Europe and the USA work at night or have rotating shifts [197]. Moreover, approximately 80% of the population of the world is exposed to light at night. This exposure makes them vulnerable to disrupted circadian rhythms [198]. Studies have reported various complications of rhythm disorders such as metabolic disorders, diabetes, obesity, cardiovascular issues, and mental health problems including anxiety and depression [199-202].

The effects of circadian rhythm disruption and aging can manifest differently in SCLC and NSCLC due to their unique biological characteristics. SCLC, known for its aggressive growth and strong association with smoking, may be particularly susceptible to circadian disruption as it relies on rapid cell cycles and DNA repair mechanisms. Circadian genes like PER1 and BMAL1, involved in DNA damage response, may exhibit altered expression in SCLC, potentially affecting the tumor’s response to therapeutic interventions such as radiation and chemotherapy, both of which interact with the circadian control of cell cycle and apoptosis [203].

NSCLC, which accounts for about 85% of LC cases, including adenocarcinoma, squamous cell carcinoma, and large cell carcinoma, exhibits a slower progression but is also influenced by aging and circadian disruptions. Studies suggest that in NSCLC, dysregulation of circadian genes like BMAL1 may contribute to oxidative stress, immune modulation, and metabolic dysregulation, all of which are exacerbated by aging. Aging-related decline in circadian regulation may therefore increase susceptibility to NSCLC by compromising cellular defenses against oxidative damage and altering immune responses, both critical in NSCLC tumorigenesis [204]. Furthermore, recent research has shown that circadian gene expression patterns in NSCLC patients can predict outcomes, suggesting that circadian rhythm maintenance might have a differential therapeutic benefit in NSCLC management [205].

The association of circadian disruption and cancer has been a hot topic of discussion among researchers. However, recent research, including findings from the International Agency for Research on Cancer (IARC), indicates that disruptions to the human circadian clock significantly raise the risk of developing various cancers, including LC [206-213]. In a study, Lou et al. examined thyroid nodules in elderly individuals with different levels of malignancy and determined that poor sleep quality and disruptions in biological rhythms are independent risk factors. They found that the expression of BMAL1 and CLOCK were significantly elevated in the group having malignant thyroid nodule, while CRY2 expression was notably lower [214-216]. In addition to the afore-mentioned evidence, animal studies have shown a link between circadian rhythms and cancer. Mice that are not naturally prone to cancer exhibit premature aging after introducing artificial mutations in BMAL1 and CLOCK genes [117, 149]. Moreover, Papagiannakopoulos has found that prolonged jet lag significantly worsens cancer development and progression in mice deficient in K-Ras and p53 [50].

As mentioned in the previous section, c-Myc proteins play a key role in circadian clock regulation [4]. Genes such as BMAL1 and PER cause a reduction in the expression of c-Myc while the tumor is progressing and developing. The deletion of these gene results in the increased expression of c-Myc as shown in a LC animal model [50]. In a study, Ramanathan et al. [217] reported that inhibition of mTOR reduces the acitivity of circadian rhythms, suggesting mTOR’s role in their biological regulation. As a central modulator of multiple tumor-related signaling pathways, mTOR is vital for growth, differentiation, cell proliferation, and survival. Abnormal mTOR activity is strongly linked to the progression of various cancers, including LC [218, 219]. In a recent study, Chen et al. found that the expression of HLF, a circadian rhythm-related gene showed reduced expression in early-relapsed NSCLC cells and was linked to early metastasis. Upregulation of HLF caused an inhibition, while silencing HLF promoted, metastasis in vivo. Reduced HLF expression also enhanced NSCLC cell growth by inducing NF-κB/p65 signaling. Genetic deletion and methylation were identified as causes of HLF down expression, suggesting HLF as a potential target for NSCLC treatment [209].

According to the circadian disruption hypothesis, exposure to light at night could potentially disturb the body’s natural circadian rhythm. This disturbance may specifically reduce the nighttime production of the pineal hormone melatonin and its release into the bloodstream [220, 221]. In a study, Vinogradova et al. [222] investigated how constant light exposure affected rats compared to a standard light/dark cycle. Starting constant light at 1 month accelerated tumor growth and shortened lifespan in both sexes. Beginning constant light at 14 months didn’t affect survival but impacted tumor growth differently in males and females. The study suggests that early-life exposure to constant light disrupts natural biological rhythms, leading to accelerated aging processes and promoting tumor formation in rats [222].

In another important study, Pariollaud et al. investigated the influence of chronic circadian disruption with chronic jetlag (CJL), in a LC mouse model. An increase in the tumor burden was observed due to chronic circadian disruption. To reveal the molecular mechanisms behind this observation, they discovered that chronic circadian disruption induced the expression of heat shock factor 1 (HSF1), a protein that is known for its pro-tumorigenic activities. Furthermore, they found that CJL disrupted the normal rhythmic movement of HSF1 into the nucleus of lung cells, leading to increased accumulation of HSF1 in the nucleus. To explore its further role in LC, they pharmacologically inhibited HSF1, which interestingly led to reduced cancer growth. This suggests that HSF1 could be a potential link between aging-induced circadian disruption and LC [223].

As mentioned in the previous sections, PER1 has been demonstrated to participate in the DNA damage repair process alongside ATM and Chk2, forming a complex with them that facilitates apoptosis following DNA damage [224-226]. Lower levels of PER1 have been implicated in impaired DNA damage repair and have been linked to the development of both lung and breast cancer [226, 227]. To check the DNA repair activity, Paz-Elizur and colleagues conducted a study involving 150 non-small cell LC patients and 143 controls. Their results demonstrated that the DNA Repair score was significantly lower in LC patients compared to controls and was associated with an increased risk of LC [228].

Block KI et al. investigated the impact of disrupted circadian rhythms in different types of cancers including late-stage LC. They identified increased cancer incidence due to nocturnal light exposure and circadian disruption and noted that untreated cancer patients suffer from disrupted sleep, mood disturbances, fatigue, and anorexia. The study investigated the therapeutic potential of adjusting the circadian clock to improve the quality of life and survival rates of cancer patients. The study concluded that manipulating the circadian clock through behavioral techniques, actigraphic monitoring, and electronic feedback can significantly improve the quality of life and survival of cancer patients [229].

PER1 and BMAL1, while commonly recognized for their regulatory functions in circadian rhythms and aging, play pivotal roles in LC at the molecular level. PER1, for instance, is intricately involved in the DNA damage response. In normal cellular processes, PER1 coordinates with ATM (ataxia-telangiectasia mutated) and Chk2 (checkpoint kinase 2) to mediate DNA repair, especially following cellular stress or DNA damage events [227]. However, in LC, reduced expression of PER1 has been observed, which leads to impaired DNA repair capabilities [230]. This impairment allows for the accumulation of genetic mutations, heightening the risk of oncogenic transformations in lung tissue. Consequently, low levels of PER1 have been linked to a higher likelihood of tumor initiation and growth in LC [231].

BMAL1, on the other hand, is crucial in maintaining oxidative homeostasis. BMAL1 controls the expression of antioxidant enzymes that mitigate reactive oxygen species (ROS) accumulation, a common contributor to cellular aging and carcinogenesis [232]. In LC, BMAL1 is often downregulated, resulting in elevated ROS levels within lung cells. The increase in oxidative stress contributes to DNA damage and inflammation, creating a favorable environment for cancer progression [233]. Furthermore, BMAL1 has a role in cell cycle regulation, acting as a checkpoint that ensures cells do not proliferate uncontrollably [234]. When BMAL1 function is compromised, as seen in various LC studies, cells bypass these checkpoints, leading to unregulated cell division and tumor growth. Thus, the dysregulation of both PER1 and BMAL1 not only reflects circadian disruption but also directly contributes to the molecular pathogenesis of LC by undermining DNA repair, increasing oxidative stress, and permitting unchecked cell proliferation.

Interest in the research of sirtuins grew exponentially when its role was revealed the longevity [235-242]. A study revealed that additional copies of S2 in Saccharomyces cerevisiae increased the lifespan by approximately 30% through preventing the formation of extrachromosomal DNA circles [243]. Now, sirtuins are known as the key regulators of aging and many research reports have documented their role in longevity. It is also reported that these sirtuins actually delay age-associated telomere attrition and enhance DNA integrity and repair mechanisms [244]. SIRT1 activates AMPK by directly deacetylating LKB1, an AMPK regulator. Furthermore, AMPK plays a role in the longevity effects of IIS, indicating that these longevity-related pathways interact closely with each other [245].

SIRT2 has been recognized as one of the key players in aging, however much of the is now focused on SIRT1 [246-259]. Sun et al. investigated to see whether induction of apoptosis and radiation sensitivity could be achieved by inhibiting Sirt1 with antisense oligonucleotides in A549 LC cells [260]. Their results showed that the inhibition of Sirt1 reduced the expression of Sirt1 in a sequence-specific and dose-dependent manner at both mRNA and protein levels. This inhibition led to increased apoptosis, reduced cell survival, and increased G1 cell cycle arrest. The antiproliferative effects of radiation were also increased by the increased acetylation of the tumor suppressor p53 and Bax expression. These results suggest that targeting Sirt1 with antisense oligonucleotides could be a potential gene therapy approach for treating LC.

Despite the reduced functions of the aging body, one of the devastating declines is linked to the central nervous system (CNS) [261-267]. Dysfunction in the CNS, affecting memory, cognitive ability, motor control, hearing and, can lead to a faster decline in one’s quality of life. Over the last few years, the risk of neurodegenerative diseases such as Alzheimer’s disease (AD), Parkinson’s disease (PD), Huntington’s disease (HD), and other aging-related diseases has risen significantly with advancing age [268-273]. In animal models of Alzheimer’s disease, specifically, the deletion of SIRT1 specifically in the brain led to a notable increase in reactive gliosis and β-amyloid plaques [274]. Deletion of SIRT1 specifically in the brain reduced the neurotoxic effects linked to mutant huntingtin protein, whereas increasing SIRT1 levels mitigated this toxicity [275, 276]. Moreover, in another mouse model with Parkinson’s disease, enhancing sirt1 expression caused a reduction in α-synuclein aggregation, decreased gliosis, and lowered mortality rates [277]. In a mouse model in which axonal degeneration was induced by injury, maintaining NAD^+^ biosynthesis and supporting sirt1 activity was crucial for preventing axonal loss after axonal transection [278, 279].

**Figure 5.**
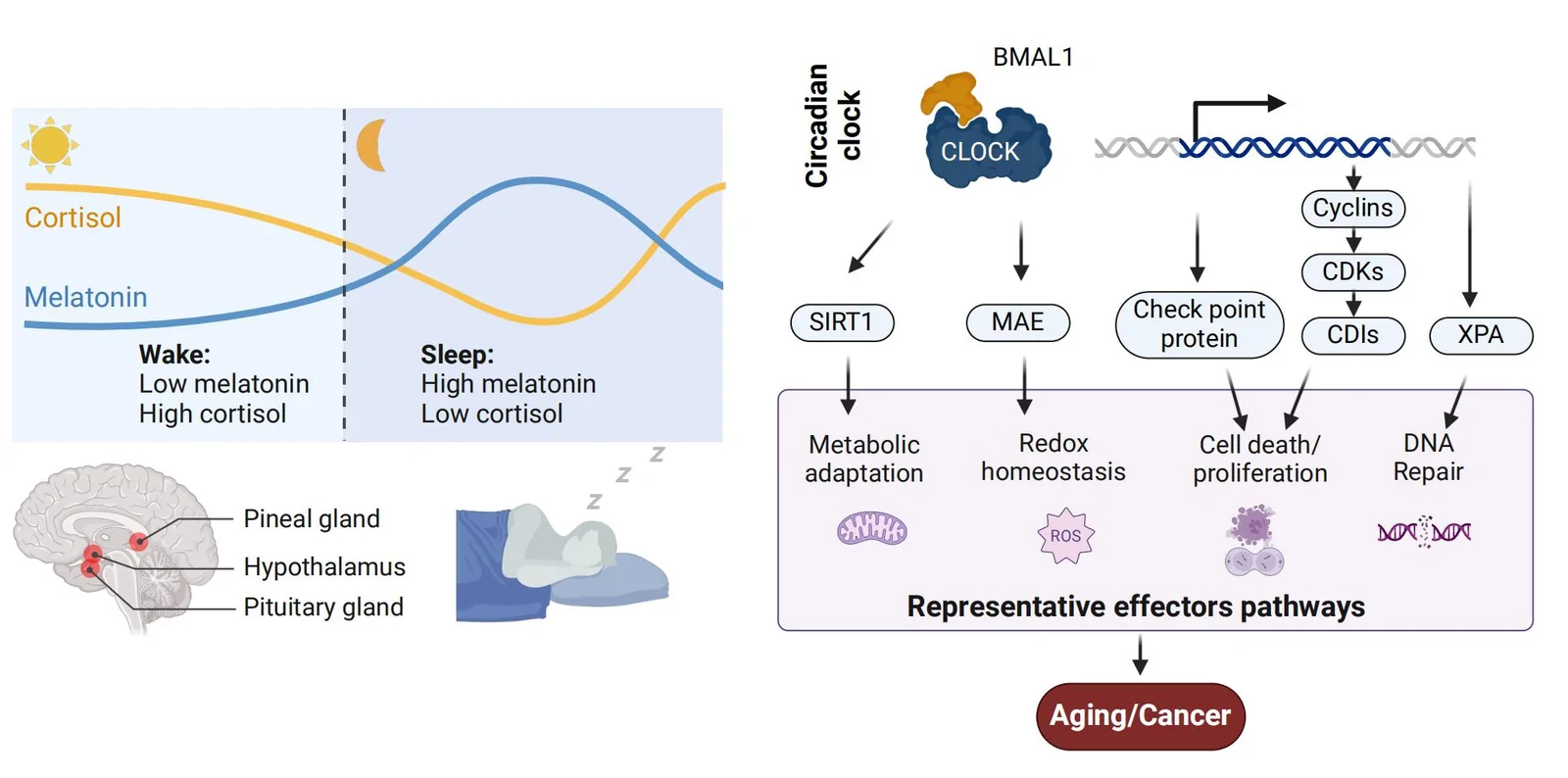
**A graphical representation of circadian rhythms and related mediators between circadian and aging/cancer**. Activation of BMAL1/CLOCK downstream molecular pathways could affect Metabolic adaptation, redox homeostasis, cell death and proliferation, and also DNA repair, which play a significant role in the progression of aging and pathogenesis of LC.

Genes associated with cell cycle control also play a very key role in controlling the circadian clock as age progresses. The cell cycle and its associated genes are also strongly linked with carcinogenesis of several cancers including LC [280, 281]. As shown in Figure 5, the circadian clock regulates ROS homeostasis, DNA repair, cell death, proliferation, and metabolism through several mechanisms including protein-protein interactions and transcriptional control mechanisms [282-290]. Accumulation of ROS leads to oxidative stress and DNA damage which consequently results in several diseases including LC [291-295].

It has been reported that ROS is increasingly regulated by BMAL1 and when BMAL1 is downregulated, that leads to the accumulation of ROS and oxidative stress. While the exact mechanisms of this regulation are not known, however, it is believed that some antioxidant enzymes could be the transcriptional targets of BMAL1 [296]. Genomic integrity and DNA repair mechanisms also play important roles in circadian clock regulation and aging. Defective repair mechanisms and loss of integrity have been associated with increased incidence of LC and tumor progression [297-301]. To conclude, the intricate interplay between circadian rhythms and aging leads to LC involving various cellular and molecular mechanisms. Key proteins such as c-Myc, PER1, BMAL1, HLF, and SIRT1 have been identified as crucial regulators within these processes.

## 6. Circadian Disruption in LC: Molecular Insights and Therapeutic Strategies

In exploring the complex relationship between circadian rhythms, aging, and LC, insights from molecular biology, clinical oncology, and chronotherapy provide a comprehensive perspective on how these processes intersect. On a molecular level, circadian genes such as BMAL1, CLOCK, and PER1 regulate DNA repair, apoptosis (Table 2), and oxidative stress responses, which are essential for maintaining cellular integrity and preventing tumorigenesis [55]. In LC, dysregulation of BMAL1 is associated with increased oxidative stress, which exacerbates age-related susceptibility to NSCLC by compromising antioxidant defenses and enhancing inflammation-a key driver of lung tumor progression [50, 51].

**Table 2:** Key Circadian Genes and Their Roles in LC Progression.

Gene	Function in Circadian Rhythm	Mechanistic Role in LC	Observed Clinical Implications
**BMAL1**	Regulates oxidative stress and DNA repair	Acts as an antioxidant modulator, reducing ROS levels; controls cell cycle and apoptosis	Low BMAL1 levels linked to increased oxidative stress, immune dysfunction, and tumor growth in NSCLC and SCLC
**PER1**	Controls DNA damage response and cell cycle checkpoints	Facilitates DNA repair by forming complexes with ATM and Chk2; critical for apoptosis	Reduced PER1 expression impairs DNA repair mechanisms, increasing mutation accumulation and LC risk
**CLOCK**	Core circadian regulator that pairs with BMAL1 to control gene transcription	Overexpression leads to continuous cell proliferation and loss of growth control	High CLOCK expressions associated with poorer prognosis in lung cancer due to disrupted cell cycle regulation
**CRY2**	Inhibits BMAL1/CLOCK complex to maintain rhythm stability	Decreased CRY2 expression linked to increased cancer cell survival and metabolic reprogramming	Lower CRY2 levels observed in aggressive lung cancers, suggesting a role in tumor metabolism and survival advantage
**c-MYC**	Interacts with circadian proteins, influencing metabolism and proliferation	Upregulates metabolic genes, aiding cancer cell adaptation to stress	Elevated c-MYC disrupts circadian homeostasis, promoting tumorigenic processes, especially in aggressive lung cancers

From a clinical oncology perspective, circadian disruptions are linked to poorer prognosis and altered responses to standard treatments in both SCLC and NSCLC. For instance, SCLC, which relies on rapid cell cycles and robust DNA repair mechanisms, shows altered expression in circadian genes such as PER1 and CLOCK, which may modulate sensitivity to radiotherapy and certain chemotherapeutic agents [52]. In NSCLC, where inflammation and immune modulation are predominant, recent studies demonstrate that restoring BMAL1 function could potentially reduce oxidative stress and support anti-tumor immunity, opening avenues for circadian-based therapies [44].

Chronotherapy, which involves timing treatment administration to align with the patient’s circadian rhythms, has shown promise in enhancing therapeutic efficacy and reducing side effects. Studies conducted on various cancers, including LC, reveal that synchronizing drug delivery with peak circadian gene expression can improve drug metabolism and reduce toxicities [302]. For example, chemotherapy agents like cisplatin and paclitaxel have shown increased efficacy when administered in alignment with circadian rhythms, suggesting potential for improved clinical outcomes in LC patients when treatment is timed to individual circadian profiles [303]. Further studies are warranted to establish personalized chronotherapy protocols that integrate patient-specific circadian markers, potentially enhancing both survival and quality of life in LC patients [304].

This multidisciplinary approach highlights the need for future research combining molecular studies, clinical trials, and chronotherapy in LC. Such research could lead to novel, personalized treatment regimens that consider the patient’s unique circadian profile, addressing the biological and clinical complexities of circadian disruptions in aging and LC.

## 7. Summary

Aging and disruption to the circadian rhythms, also known as biological clocks, are complex and bi-directional processes. The process of aging ultimately leads to circadian disruption and changes in the circadian clocks promote aging. Both processes have a potential role in the progression and pathogenesis of LC. Aberration in biological clocks results in changes in the physiological functions that lead to progressive aging and promote the susceptibility of LC. These changes are mostly accompanied by reduced melatonin and disturbed sleep/wake cycles. Various genes and molecular pathways including SIRT1, BMAL1, PER1, and c-Myc play a potential role in chronic circadian disruption, aging, and LC. Finding novel therapeutic targets such as epigenetic regulations of several potential genes may pave the way for new insights into the management of circadian disruptions, progressive aging, and LC development.

In examining the interplay between circadian rhythms, aging, and LC, we recognize discrepancies in the literature regarding the expression and functional roles of core circadian genes, such as BMAL1, PER1, and CRY2-across different LC subtypes and patient demographics. While some studies suggest a direct correlation between disrupted circadian regulation and enhanced tumorigenic potential, others report variable outcomes that depend heavily on the experimental models, circadian parameters assessed, and the underlying cancer subtype. For instance, in NSCLC, dysregulated BMAL1 expression has been associated with increased oxidative stress and immune modulation, yet contrasting data highlight variable effects in SCLC, where circadian influence on DNA repair mechanisms may be more pronounced.

These discrepancies suggest several promising avenues for future research. First, longitudinal studies are needed to delineate the role of specific circadian genes in LC progression across diverse patient populations, considering variables such as age, genetic background, and environmental factors. Second, mechanistic studies focused on how circadian disruptions impact cell cycle regulation, DNA repair fidelity, and immune response in distinct LC subtypes could yield insights into subtype-specific vulnerabilities. Additionally, exploring targeted interventions that restore circadian rhythm functionality-such as BMAL1 activation or PER1 stabilization-might offer novel therapeutic strategies. Addressing these research directions could provide a more comprehensive understanding of the circadian-cancer nexus and support the development of chronotherapy approaches tailored to

LC subtypes and patient-specific circadian profiles. The insights gained from circadian rhythm research in LC have profound implications for both clinical practice and public health policy (Table 3). Clinically, the integration of circadian biology into therapeutic protocols, particularly through chronotherapy, could optimize treatment outcomes by aligning drug administration with the patient’s biological rhythms. For instance, chemotherapeutic agents such as cisplatin and paclitaxel may achieve heightened efficacy and reduced toxicity when administered during phases that correspond to peak expression of DNA repair and apoptotic regulatory genes in tumor cells [302, 303]. From a public health perspective, addressing environmental factors that contribute to circadian disruption, such as shift work and exposure to artificial light at night, could play a preventive role in cancer risk management. Policy initiatives might consider recommendations to minimize night shift work where possible, as well as guidelines to manage light exposure in occupational and urban settings, thereby mitigating the adverse health effects associated with circadian misalignment [43, 51]. Furthermore, public education on the importance of stable sleep-wake cycles could foster greater awareness of circadian health, potentially reducing the population-wide burden of circadian-disruption-associated cancers [305]. The translation of these findings into clinical guidelines and public policy requires interdisciplinary collaboration, aiming to integrate circadian principles into cancer prevention strategies, workplace regulations, and individualized patient care plans. Such initiatives could help lower the incidence of circadian-related cancers and support better outcomes for LC patients by facilitating rhythm-informed therapeutic interventions.

**Table 3:** Translational Applications of Circadian Findings in LC for Clinical Practice and Public Health.

Application Area	Proposed Strategies	Expected Benefits
**Chronotherapy**	Aligning chemotherapy and radiotherapy with circadian rhythms to maximize drug efficacy	Potential to increase therapeutic impact, reduce adverse effects, and improve patient outcomes
**Night Shift Regulation**	Adjusting shift timings and offering protective guidelines to minimize circadian disruptions	Reduces health risks related to circadian misalignment, potentially decreasing cancer risk among night-shift workers
**Public Education**	Promoting awareness of circadian health and stable sleep patterns for cancer prevention	Enhances public health by reducing circadian disruption-related cancer risks, especially in high-risk populations
**Targeted Therapies**	Developing circadian gene modulators (e.g., BMAL1 activators, PER1 stabilizers)	Personalized treatments aligned with patient circadian profiles, potentially improving therapeutic precision
**Light Pollution Control**	Establishing guidelines on artificial lighting to maintain natural circadian rhythms	Reduces light exposure at night, potentially lowering population-wide circadian disruption and associated cancer risks

Despite significant advances in understanding the role of circadian rhythms in LC, several limitations constrain the field. One notable challenge is the complexity and variability in circadian gene expression across different LC subtypes. Studies have shown that genes like BMAL1 and PER1 exhibit divergent effects depending on the subtype, but there is a lack of comprehensive, subtype-specific analyses that could clarify these mechanisms. Future research should focus on large-scale, longitudinal studies that track circadian gene expression patterns in diverse LC subtypes, providing a clearer picture of subtype-specific roles. Another limitation lies in the translation of circadian biology insights into clinical settings. While chronotherapy holds promise, there is limited guidance on integrating circadian-based treatments with existing LC therapies like chemotherapy and immunotherapy. Future research could involve clinical trials to assess the efficacy of administering these treatments according to circadian timing, potentially enhancing patient outcomes and minimizing side effects. Lastly, the influence of aging on circadian regulation in LC remains underexplored. Aging-related circadian disruptions might exacerbate LC susceptibility and progression, yet studies specifically examining elderly LC patients are sparse. Research efforts could be directed toward understanding the interplay between aging, circadian rhythms, and LC, ultimately informing preventive measures and tailored treatment options for elderly populations.
